# The Inhibitory Effect of Peptide Hydrolysate of Type I Collagen Derived from Pig Skin on Melanogenesis in B16F10 Melanoma Cells

**DOI:** 10.3390/biom15020220

**Published:** 2025-02-03

**Authors:** Jialin Zheng, Dandan Xu, Tianduo Li

**Affiliations:** 1 School of Chemistry and Chemical Engineering, University of Jinan, Jinan 250024, China; 201811100026@stu.ujn.edu.cn; 2 Experimental Center, Shandong University of Traditional Chinese Medicine, Jinan 250355, China; 3 Shandong Provincial Key Laboratory of Molecular Engineering, School of Chemistry and Chemical Engineering, Qilu University of Technology (Shandong Academy of Sciences), Jinan 250353, China

**Keywords:** collagen peptide, bioactive peptide, melanin inhibition, tyrosinase inhibition

## Abstract

Collagen peptides, as a natural source of peptides, possess multiple advantages such as anti-aging, anti-inflammatory properties, tissue repair, and the ability to inhibit melanin production. In this study, type I collagen extracted from pig skin was hydrolyzed with 1% and 3% hydrochloric acid, yielding collagen peptides CPH1 and CPH3. The melanin content and tyrosinase activity in B16F10 cells were compared via direct and paracrine action when CPH1 and CPH3 were used to interfere with melanogenesis. It was found that CPH3 significantly inhibited melanogenesis in B16F10 through the paracrine action involving HaCaT keratinocytes. The intracellular melanin content was measured at 65.23 ± 1.30%, and the mRNA levels of tyrosinase and microphthalmia transcription factor in cells were 55.77 ± 6.09% and 50.70 ± 8.18% of the negative control, respectively. Furthermore, pigment deposition assays in zebrafish showed that, at a concentration of 1.0 mg/mL, CPH3 significantly inhibited melanogenesis compared to the negative control. Finally, tyrosinase inhibitory peptides were identified from CPH3 through peptide segment sequence identification and molecular dynamics simulation. The peptides of Nona-AGPPGFPGA, Octa-APGPVGPA, and Octa-GLPGPPGP have a double effect on the inhibition of tyrosinase and melanin content in B16F10 cells.

## 1. Introduction

Collagen stands as one of the most abundant and crucial structural proteins found in both humans and animals. Its distinctive triple-helix structure, coupled with ultra-high molecular weight, low immunogenicity, and commendable biocompatibility, renders collagen highly sought after in the realm of tissue engineering materials [1,2]. Presently, a spectrum of collagen products with varying structures, molecular weights, active sites, and substrates has emerged. Notably, collagen of smaller molecular weight finds predominant application in dermatology and skincare products, while medium molecular weight variants feature prominently in the development of second-class medical devices. On the other hand, large molecular weight collagen, following cross-linking and modification, serves as a potential substitute material in tissue engineering. Within the skin, fibroblasts are responsible for collagen production, which is crucial for supporting facial muscles and maintaining skin elasticity. However, with advancing age, the synthesis rate of collagen diminishes significantly, precipitating a notable loss of collagen content and subsequent skin laxity, dryness, and the emergence of age spots [3]. Various methods, including acid, alkaline, enzymatic, and microwave approaches, are employed for collagen biodegradation, yielding collagen peptides characterized by excellent water solubility, reduced molecular weight, and heightened biological activity [4,5,6,7].

Collagen polypeptide possesses remarkable water absorption and hydrating properties, forming a protective film on the skin surface that effectively mitigates epidermal water loss. Hydrogels compounded or modified with other polymer materials exhibit additional attributes such as moisturizing, anti-inflammatory effects, and the promotion of cell proliferation [8,9,10,11]. They also play a role in scavenging peroxides and harmful free radicals within cells, consequently reducing cellular oxidative stress [12,13,14,15]. Additionally, collagen peptides exhibit the potential to inhibit melanin production, a process crucial in mitigating the effects of ultraviolet (UV) radiation, a primary exogenous factor contributing to excessive melanin deposition in human skin. Increased UV radiation prompts keratinocytes to secrete elevated levels of melanin, subsequently upregulating microphthalmia-associated transcription factor (MITF) expression and enhancing tyrosinase (TYR) activity, thereby leading to excessive melanin secretion. TYR, functioning as a rate-limiting enzyme in melanin biosynthesis, is pivotal in regulating this process. Notably, known collagen peptides capable of inhibiting melanin production predominantly originate from fish sources. Zu et al. [16] isolated a triple-helix collagen peptide with a molecular weight ranging from 6096.68 to 9513.70 Da from silverfish scales. Notably, hydrophobic and alkaline amino acids constitute a significant portion of the peptide segment composition, accounting for 32.15% and 13.12%, respectively. Bioactivity predictions suggest that peptide fragments, comprising hydrophobic amino acids like proline and alanine, along with alkaline amino acids such as arginine, can bind to TYR and diminish its activity, thereby manifesting whitening potential [17,18,19]. Collagen peptides extracted from fish bones exhibit potent inhibitory effects on intracellular melanin and TYR activity, effectively reducing skin oxidation, inflammation, and UV-induced pigment deposition damage [20,21].

This study successfully obtained two molecular weight distributions of pig skin type I collagen peptides (CPH1 and CPH3) by adjusting the intensity of H+, and it delves into the influence of collagen-peptide molecular weight and mechanism of action on melanin production. Findings reveal that CPH3 demonstrates the capability to inhibit melanin production in mouse melanoma cells (B16F10) and to diminish intracellular TYR activity via a paracrine action involving keratinocytes (HaCaT). The collagen polypeptide with TYR activity was screened through amino acid sequence analysis of CPH3 and in vitro cell experiments. These insights offer novel perspectives and a theoretical framework for the advancement of collagen-peptide-based whitening products and the management of pigmentation lesions.

## 2. Materials and Methods

### 2.1. Extraction of Type I Collagen

Fresh pig skin was bought from a meat supermarket and thoroughly degreased using NaOH solution, organic solvent (chloroform:anhydrous ether = 2:1 volume ratio), and anhydrous ethanol. After that, 10% NaCl solution was used to stir for 24 h to remove impurity proteins. A total of 1% pepsin of pig skin mass was added to 0.5 M acetic acid and stirred at 4 °C for 48 h to hydrolyze. After centrifuge, the supernatant was repeatedly mixed with 1 mM NaCl to salt out three times, which were then dissolved in 1% glacial acetic acid and placed into a dialysis bag. The solution was dialyzed in 0.1% acetic acid solution for 3 days and finally freeze-dried for 24 h to obtain collagen.

### 2.2. Preparation of Collagen Peptide

Type I collagen was extracted from pig skin using the acid enzyme method. Newly purchased fresh pig skin had its oil removed and was washed before being cut into small squares. A total of 0.075 mm NaOH solution was mechanically stirred in a room-temperature water bath for 24 h, rinsed, and drained. Then, the collagen was placed in a mixture of chloroform and anhydrous ether (V_1_:V_2_ = 2:1) for 24 h. Anhydrous ethanol was then stirred in until there was no odor, and then it was air-dried. Then, 10% NaCl solution was stirred in for 24 h and centrifuged for precipitation. A total of 0.5 M glacial acetic acid was prepared, and porcine stomach mucose (1:30,000), which was 1% of pig skin mass, was added to the pig skin and stirred at low temperature for 48 h. After centrifugation, salting out, and multiple precipitations, the product was dissolved and precipitated with glacial acetic acid and put into a dialysis bag, which was then dialyzed in glacial acetic acid solution and distilled water. Freeze and freeze dry after dialysis.

Following freeze-drying, the collagen was dissolved in 1%, 2%, and 3% hydrochloric acid and subjected to degradation in an 80 °C water bath for 4, 6, and 8 h, yielding a 1 mg/mL collagen-peptide solution. Upon completion of the reaction, the acidic solution was neutralized with sodium hydroxide and subsequently placed in a dialysis bag with a molecular weight cutoff greater than 500 Da (obtained from Shanghai Yuanye Biotechnology Co., Ltd., Shanghai, China) for 24 h. Following dialysis, the solution was freeze-dried and then dissolved in a culture medium, resulting in a 50 mg/mL collagen-peptide reserve solution.

### 2.3. Planar Bounded Point Set Model for Calculating the Length of Collagen Single Molecules

Using MATLAB (R2018) to construct the minimum bounding circle of a planar point set.

$$x_{1}=\min\{x\left|(x,y)\in D\right.\},x_{2}=\max\{x\left|(x,y)\in D\right.\},y_{1}=\min\{y\left|(x,y)\in D\right.\},y_{2}=\min\{y\left|(x,y)\in D\right.\}.$$

The minimum axis-oriented bounding box for *D* is:

$$D_{R}=\{(x,y)\left|x_{1}\leq x\leq x_{2}\right.,\left|y_{1}\leq y\leq y_{2}\right.\}.$$

Based on the linear characteristics of collagen molecules, for the bounded point set *D* on a plane, firstly, the two farthest points were selected on the centerline, and an initial circle with the diameter of the connecting line was constructed between these two points. If this initial circle contained all the points in *D*, then the circle was the minimum enclosing circle of the point set *D*. Otherwise, the point *D* outside the circle that is farthest from the center of the circle was searched for, a new circle using these three points as points on the circle was constructed, and then the farthest point first asymptotic algorithm was used to explore the subsequent minimum enclosing circle, ultimately the minimum enclosing circle of the centerline was obtained.

### 2.4. Characterization of Type I Collagen/Collagen Peptide

Sodium dodecyl sulphate-Polyacrylamide gel electrophoresis (SDS-PAGE): 1 mg collagen sample was dissolved in 1 mL sample solution, mixed evenly, and boiled for 5 min. It was marked with a broad-spectrum protein marker (11–245 kDa), concentrated with 5% concentrate glue, 7% separation glue, and 20 mA current, and then separated with 40 mA current. After separation, the samples were stained and decolorized.

Fourier transform infrared spectroscopy (FTIR, TENSOR II, Bruker, Karlsruhe, Germany) was used to analyze the characteristic absorption peaks of collagen-specific groups at 500–4000 cm^−1^.

Circular dichroism spectrometer (CD, Chirascan, Applied Photophysics, Leatherhead, UK): Preparation of collagen solution with a mass concentration of 10 μg/mL and fixed measurement wavelength range of 190–260 nm.

Matrix-assisted laser desorption ionization time-of-flight mass spectrometry (MALDI-TOF MS, New ultrafleXtreme, Bruker, Billerica, MA, USA): Collagen solution was mixed with erucic acid as the medium and then placed on the target plate for natural drying. Bovine serum albumin was used as the standard sample for calibration.

Atomic force microscopy (AFM, Cypher ES, Oxford Instruments, Abingdon, Oxfordshire, UK): The freeze-dried collagen was dissolved in 0.05 mM glacial acetic acid to obtain 1 mg/mL collagen reserve solution and diluted 1000 times with Tris-HCl at pH = 6.8–7.2. The collagen diluent of Taked 10 μL was absorbed on the surface of the newly peeled mica for 2 min, rinsed with ultra-pure water for 30 s, and then dried with nitrogen. The probe type was AC160TS, the frequency in the gas phase was 260–300 kHz, and the spring constant was 26 N/m.

### 2.5. Cell Culture

Mouse melanoma cells (B16F10) and human keratinocytes (HaCaT) were cultured in high-glucose DMEM medium (Procell Life Science & Technology Co., Ltd., Wuhan, China), supplemented with 10% fetal bovine serum (Procell Life Science & Technology Co., Ltd., Wuhan, China), and a mixture of 1% penicillin and streptomycin (Solarbio Life Science Co., Ltd., Beijing, China). The incubator environment was maintained at 37 °C with 5% CO_2_.

### 2.6. Cell Viability Assay

1 × 10^4^ cells were seeded per well in a 96-well plate, the original culture medium was discarded after cell attachment, and collagen peptide dissolved in 10% FBS-DMEM was added. After 48 h, 10 μL CCK8 reagent with a concentration of 5 mg/mL was added to each well (Shanghai Yuanye Biotechnology Co., Ltd., Shanghai, China). After incubation in a 37 °C incubator for 2 h, the absorbance value at 450 nm was measured by an enzyme-linked immunosorbent assay (BioTek Cytology 5, Winooski, VT, USA), and cell viability was calculated according to the following formula:

$$\mathrm{Cell}\;\mathrm{survival}\;\mathrm{rate}\;(\%)=\frac{{(OD}_{s}-{\;OD}_{b)}}{{(OD}_{c}-{OD}_{b})}\times 100\%$$

In the formula, ‘*s*’, ‘*b*’, and ‘*c*’ represent the sample group, blank group, and control group, respectively.

### 2.7. Melanin Synthesis Assay

In the non-contact cell coculture method, the impact of HaCaT culture supernatant on melanogenesis in B16F10 cells was investigated. Initially, 5 × 10^5^ HaCaT cells were seeded into each well of a 6-well plate. Once the cells adhered to the surface, the original culture medium was replaced with 10% FBS-DMEM containing dissolved collagen peptides. After 48 h, the supernatant was harvested and transferred to a separate 6-well plate containing 5 × 10^5^ B16F10 cells, where it was incubated for an additional 48 h. Subsequently, 100 μL of supernatant was collected from each well and transferred to a 96-well plate. The melanin content in the supernatant was quantified by measuring the absorbance at 405 nm using an enzyme-labeled instrument. Following the measurement, the remaining supernatant was discarded, and 250 μL of NaOH (containing 10% DMSO) at a concentration of 1M was added to each well. The plate was then subjected to an 80 °C water bath for 30 min. After vortexing, 100 μL of supernatant was transferred to a new 96-well plate, and the absorbance at 405 nm was measured.

$$\mathrm{Melanin}\;\mathrm{content}\;(\%)=\frac{{(OD}_{s})}{{(ODAVG}_{c})}\times 100\%$$

In the formula, ‘*s*’ denotes the average values of the sample group, and ‘*AVGc*’ represents the average values of the control group.

### 2.8. Inhibition of Tyrosinase Activation

In the non-contact cell coculture method, the impact of HaCaT supernatant on TYR activity in B16F10 cells was investigated. Initially, 5 × 10^5^ HaCaT cells were seeded into each well of a 6-well plate. Once the cells adhered to the surface, the original culture medium was replaced with 10% FBS-DMEM containing dissolved collagen peptides. After 48 h, the supernatant was transferred to a separate 6-well plate containing 5 × 10^5^ B16F10 cells, and the cells were cultured for an additional 48 h. Following cultivation, the supernatant was discarded, and 250 μL of Triton X-100 (Shanghai Yuanye Biotechnology Co., Ltd., Shanghai, China) was added to each well at 4 °C for 30 min. The mixture was then centrifuged at 12,000 rpm for 20 min. Subsequently, 100 μL of supernatant was transferred to a 96-well plate, and 10 μL of L-DOPA (Merck, Darmstadt, Germany) at a concentration of 20 mM was added. The plate was incubated in a 37 °C incubator for 4–8 h, and the absorbance at 405 nm was measured using an enzyme-labeled instrument reader.

### 2.9. B16F10 Fontana–Masson

2 × 10^4^ HaCaT cells were seeded onto a 24-well glass slide culture plate and treated with collagen peptides for 48 h. Following this, the supernatant was collected and used to culture B16F10 cells for an additional 48 h. Subsequently, 4% neutral paraformaldehyde was added to fix the cells, and Fontana–Masson staining (Abcam, ab150669, Cambridge, MA, USA) was performed.

### 2.10. Western Blot Analysis

The B16F10 cells in the well plate were lysed using RIPA lysis buffer (Epizyme Biomedical Technologies Co., Ltd., Shanghai, China), and the supernatant was collected after centrifugation for the detection of intracellular protein content using a BCA assay (Epizyme Biomedical Technologies Co., Ltd., Shanghai, China). The SDS-PAGE gel concentration was set at 10%, with 30 μg of sample loaded per lane. Primary antibodies against tyrosinase and MITF were procured from Abcam (AB Co., Ltd., Cambridge, MA, USA), while the β-actin antibody was obtained from Cell Signaling Technology (CST Co., Ltd., Boston, MA, USA). The secondary antibody, goat anti-rabbit, was purchased from Abclonal Co., Ltd., Wuhan, China. ECL Plus (Epizyme Biomedical Technologies Co., Ltd., Shanghai, China) chemiluminescence was utilized to visualize the immune response bands.

### 2.11. Real-Time Quantitative-Polymerase Chain Reaction

RNA extraction was conducted using the kit from Accurate Biotechnology Co., Ltd., Changsha, China, followed by reverse transcription performed at a consistent RNA concentration of 1000 ng (TianGen Biotechnology Co., Ltd., Beijing, China). The fluorescence quantitative PCR assay kit (TianGen Biotechnology Co., Ltd., Beijing, China) was employed to monitor the fluorescence change curves of each gene. The threshold cycle (Ct), representing the number of cycles at which significant statistical increases in fluorescence first appeared for each gene, was normalized to the β-Ct of actin. The relative expression was performed by the 2^ΔΔCt^ method to calculate the differences between genes.

TYR gene forward primer 5′-GGCCAGCTTTCAGGCAGAGGT-3′, MITF gene forward primer 5′-ACTTTCCCTTATCCATCCACC-3′, reverse primer 5′-TGAGATCCAGTTTGTCGTACA-3′; β-Positive primer 5′-CGAGGGAATCGTGTGACATTAAGGAGA-3′ for actin gene, and reverse primer 5′-CGTCAT-ACTCCTGTGATCCAATCTGC-3′. The real-time fluorescence quantitative PCR instrument model was Roche Light Cycler 480 II.

### 2.12. Zebrafish Model

Wild-type (AB) zebrafish embryos were selected 24 h after fertilization. Subsequently, collagen-peptide drugs were administered for 48 h, and the mortality rate and developmental status of zebrafish larvae were assessed. The distribution of melanin in the zebrafish model was observed using a stereo fluorescence microscope (Leica M205FA, Wetzlar, Germany), and the results were analyzed using statistical software Image J (Java 1.8.0).

### 2.13. Identification of the CPH3 Peptide Sequence

CPH3 was dissolved in Nano-LC mobile phase A (0.1% formic acid/water), and the sample was uploaded onto a nanoViper C18 precolumn (3 μm, 100 Å) in a suitable volume, followed by a 20 μL volume rinse for desalting. The liquid phase was an Easy nLC 1200 nL liquid phase system (ThermoFisher, Waltham, MA, USA), and the samples were preserved by desalting on the precolumn before separation on an analytical column, which was a C18 reversed-phase column (Acclaim PepMap RSLC, Waltham, MA, USA, 75 μm × 25 cm C18–2 μm 100 Å), and the gradient used in the experiments was 30 min in mobile phase B (80% acetonitrile, 0.1% formic acid) increased from 5% to 38% in 30 min. The mass spectra were performed on a ThermoFisher Q Exactive system (ThermoFisher, USA) coupled with a nanoliter-spray Nano Flex ion source (ThermoFisher, USA), with a spray voltage of 1.9 kV and an ion transfer tube heated at 275 °C. The mass spectra were scanned in an information-dependent manner. The mass spectrometry was performed in the information-dependent acquisition mode (DDA, Data Dependent Analysis), with a primary mass spectrometry scanning resolution of 70,000, a scanning range of 350–2000 m/z, and a maximum injection time of 100 ms. A maximum of 20 secondary spectra with charges ranging from 2+ to 5+ were acquired per DDA cycle, and the maximum injection time of the secondary mass spectrometry ions was 50 ms. The collision chamber energy (high-energy collision-induced dissociation, HCD) was set to 28 eV for all precursor ions, and the dynamic exclusion was set to 25 s.

Raw data files collected by mass spectrometry were processed and retrieved for analysis using PEAKS Online 11 (Bioinformatics Solutions Inc., Waterloo, ON, Canada) software. The database was the target protein sequence downloaded from UniProt, and the search parameters were set as follows: mass tolerance of 10 ppm for primary mass spectrometry, 0.03 Da for secondary mass spectrometry, and enzyme set to none; variable modifications: protein N-terminal acetylation (Protein N-term), asparagine/glutamine deamidation (Deamidation (NQ)), and methionine oxidation (Oxidation (M)), glutamate pyroglutamylation (Pyro-glu), glutamine pyroglutaminyl cyclization (Pyro-Gln), hydroxyproline modification (hydroxyproline).

### 2.14. Molecular Docking

To construct a three-dimensional structure model of mouse tyrosine (mTYR), the protein sequence of mouse tyrosinase (Uniprot ID: P11344) was obtained through the UniProt server the mTYR model was constructed with YASARA software (version 25.1.13.) [22]. The position of Zn2+ in the crystal structure of human tyrosinase-associated protein 1 (hTYRP1) (PDB ID: 5M8T) was taken as a reference. The position of copper ion in mTYR was regarded as a parallel relationship. Based on the above, the position of Cu^2+^ was determined [23,24].

The data were processed using Python’s Pandas library (version 2.1.2), and chemoinformatic processing was performed using RDKit (version 2024.03.5). SMILES format of peptides was generated, the 3D structures of the molecules were generated, and energy was minimized using the MMFF94 force field by the AllChem module of RDKit. Molecular docking was carried out by AutoDock Vina software (vina 1.2.0) with docking parameters set as docking center coordinates (8.547, −3.481, −7.663) and docking box dimensions (30, 30, 30). The resulting optimal ligand conformation was further subjected to two-dimensional interaction analysis using LigPlus software (ligplot+ 2.2) to reveal the mode of interaction between the peptide and the target protein tyrosinase, including hydrogen bonding, hydrophobic interactions, etc.

### 2.15. Statistical Analysis

The data obtained from the experiment were subjected to statistical analysis using the standard error of the sample mean (S.E.M). GraphPad Prism software (version 8.0.2) was used for statistical analysis. Student’s *t*-test was used for pairwise comparison between two conditions. A significance threshold of *p* < 0.05 was applied to determine statistical significance in all statistical analyses.

## 3. Results

### 3.1. Structural Characterization of Collagen

**Figure 1:**
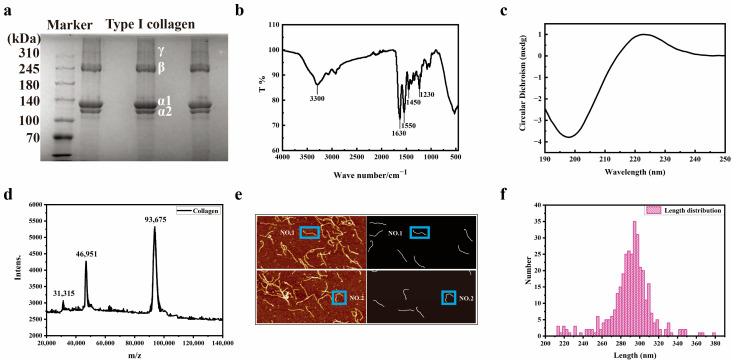
Characteristic spectrum of type I collagen. (**a**) SDS-PAGE, (**b**) FTIR-ART, (**c**) CD, and (**d**) MALDI-TOF MS of type I collagen. (**e**) The morphology of type I collagen molecules by AFM. (**f**) The minimum enclosing circle of a planar point set is used to calculate the length of type I collagen molecules.

As shown in Figure 1a, the type I collagen gel electrophoresis spectrum can be observed as a γ chain (entangled by three peptide chains), β chain (entangled by two peptide chains), α1 chain, and α2 chain. The original data of the SDS-PAGE image can be seen in Supplementary Figure S1. The content of the α1 chain is approximately two times the size of the α2 chain. By combining the molecular weight of the marker protein, it can be determined that the protein is type I collagen. Infrared spectroscopy is an important method for collagen analysis, which is used to analyze the characteristic absorption of specific collagen groups. As shown in Figure 1b, the absorption peak at 3300/cm^−1^ is an amide A band with N-H stretching vibration and the presence of hydrogen bonds. The amide I band is located at the absorption peak position of 1630/cm^−1^, with C=O stretching vibration. The amide II band exhibits N-H bending vibration at the absorption peak position of 1550/cm^−1^. The peaks near 1450/cm^−1^ to 1230/cm^−1^ in the amide III band indicate the integrity of the triple helix of collagen. Figure 1c shows the circular dichroism spectrum of collagen, with a negative peak at 193 nm and a positive peak at 220 nm. The ratio of positive peak intensity to negative peak intensity is 0.1247, indicating that the collagen triple-helix structure has been well maintained, which is consistent with the typical circular dichroism peak type of collagen triple-helix structure [25]. Due to the linear supramolecular structure of collagen molecules, MALDI-TOF was used to identify the molecular weight of collagen. After calibration with bovine serum albumin, H-bonds between peptide chains were broken under high-energy laser assistance. As shown in Figure 1d, the three peaks represent [M+H]^+^, [M+2H]^2+^, and [M+3H]^3+^, respectively, with a single chain molecular weight of 93,674 Da for collagen.

A total of 25 sets of collagen molecule gas-phase morphology images were obtained using AFM, and 348 sets of collagen molecule morphology were statistically analyzed. Each imaging group contains two images. One is a 0° scan, and the other is a 90° scan. A computer program was written using MATLAB to automatically extract the centerline of collagen molecules. Figure 1e shows the length distribution of collagen molecules, with centerline lengths of 299.0 nm and 296.4 nm for collagen molecules NO.1 and NO.2, respectively. According to statistics as shown in Figure 1f, the length of collagen molecules is mainly between 280 and 320 nm (the theoretical length of collagen molecules is 300 nm). The length pattern of collagen molecules is 297.3 nm.

### 3.2. Structural Characterization of Collagen Peptide

**Figure 2:**
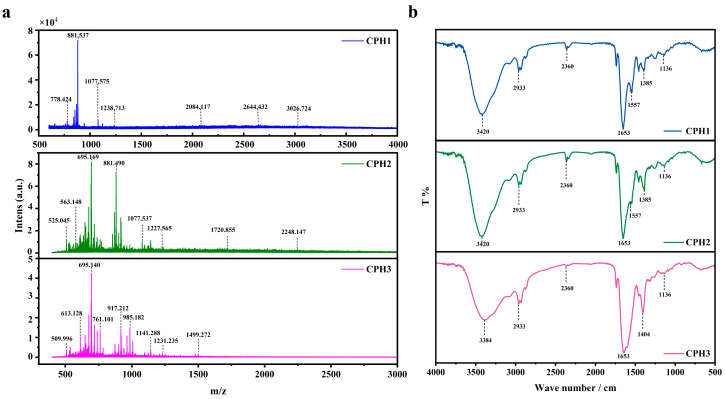
Characteristic spectrum of type I collagen peptide. (**a**) MALDI-TOF MS and (**b**) FTIR of CPH1, CPH2, and CPH3.

This study initially employed the MALDI-TOF MS method to determine the molecular weight of type I collagen hydrolysates treated with 1%, 2%, and 3% HCl, as depicted in Figure 2a. The molecular weights of CPH1, CPH2, and CPH3 gradually decreased and no longer decreased with increasing HCl/increasing temperature. As shown in Figure 2b, the characteristic peaks of 1550 cm^−1^ in the amide II band and 1230–1250 cm^−1^ in the amide III bands of CPH1 and CPH3 disappeared, indicating the loss or alteration of α-helical structure of collagen polypeptides. At this time, the characteristic peak of the amide II band 1450 cm^−1^ is blue-shifted, indicating the conformational stability of CPH1, CPH2, and CPH3 [26,27].

### 3.3. Effects of CPH1 and CPH3 on B16F10 and HaCaT Cells Viability

**Figure 3:**
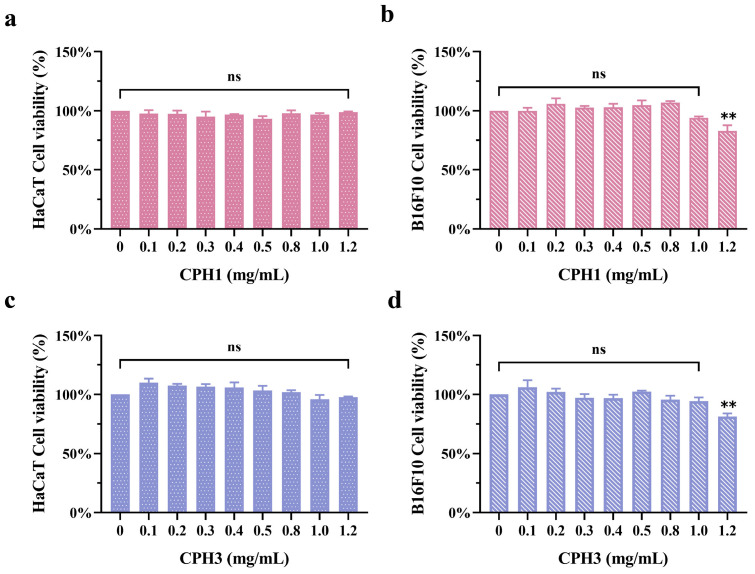
Cytotoxicity of CPH1 and CPH3. (**a**) The effects of CPH1 on the viability of HaCaT and (**b**) B16F10 cells. (**c**) The effects of CPH3 on the viability of HaCaT and (**d**) B16F10 cells, ** *p* < 0.01, versus negative control group, ns: no significant, *n* = 3.

To verify the effect of collagen-peptide molecular weight on TYR and melanin production, we compared the effects of CPH1 and CPH3 on TYR and melanin production. Following this, the impact of CPH1 and CPH3 on the viability of HaCaT and B16F10 cells was investigated. Figure 3a,b illustrates that upon treating HaCaT and B16F10 cells with CPH1 for 48 h, no significant toxic effects on HaCaT were observed within the concentration range of 0–1.2 mg/mL. However, at a peptide concentration of 1.2 mg/mL, cytotoxicity was evident in B16F10 cells. As shown in Figure 3c,d, CPH3 has no significant effect on HaCaT cells in the concentration range of 0–1.2 mg/mL. When the concentration is higher than 1.0 mg/mL, it has certain cytotoxicity on B16F10 cells. These findings suggest that collagen-peptide concentrations ranging from 0.1 mg/mL to 1.0 mg/mL can be utilized for subsequent experiments.

### 3.4. Effects of CPH1 and CPH3 on Cellular Melanogenesis

**Figure 4:**
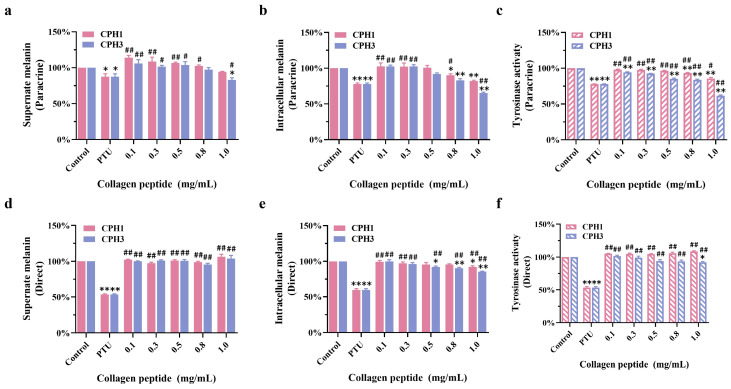
The effects of CPH1 and CPH3 on melanin and TYR. (**a**) The content of melanin in extracellular supernatant of B16F10 under paracrine action. (**b**) The content of intracellular melanin in B16F10 under paracrine action. (**c**) TYR activity in B16F10 under paracrine action. (**d**) The content of melanin in extracellular supernatant of B16F10 under direct action. (**e**) The content of intracellular melanin in B16F10 under direct action. (**f**) TYR activity in B16F10 under direct action. * *p* < 0.05, ** *p* < 0.01, versus negative control group. # *p* < 0.05, ## *p* < 0.01, versus positive control group (PTU: 1-Phenyl-2-Thiourea, 50 μM), *n* = 3.

Figure 4a–c illustrates the effects of CPH1 and CPH3 on B16F10 melanin and TYR activity under HaCaT paracrine action, respectively. The findings indicate that collagen peptides can attenuate B16F10 melanin production through the paracrine influence of HaCaT. Specifically, the melanin content in the supernatant of B16F10 cells under the influence of CPH1 and CPH3 was recorded as 94.34 ± 0.54% and 82.96 ± 3.07%, respectively. Similarly, the intracellular melanin content was reduced to 81.84 ± 1.41% and 65.23 ± 1.30% with CPH1 and CPH3 treatment, respectively. The effect of CPH2 and unhydrolyzed type I collagen on cellular melanin production is shown in Supplementary Figure S2. Moreover, CPH1 treatment resulted in an intracellular TYR activity of 85.53 ± 1.66%, whereas CPH3 treatment yielded an intracellular TYR activity of 61.50 ± 1.15% (Data shown in Table 1).

However, as depicted in Figure 4d–f, direct application of CPH1 and CPH3 on B16F10 cells did not significantly alter the melanin content in the supernatant compared to the negative control. Additionally, the inhibitory effect on intracellular melanin was weak, with values of 91.94 ± 1.81% and 85.82 ± 0.83%, respectively. Furthermore, when collagen peptides directly acted on B16F10 cells, there was no notable difference in the effect of CPH1 on TYR activity compared to the negative control. Conversely, CPH3 exhibited a lower degree of inhibition on TYR activity, with a TYR activity of 92.09 ± 1.17%.

### 3.5. Effects of CPH3 on B16F10 Fontana–Masson Staining and Relative Protein Expression

**Figure 5:**
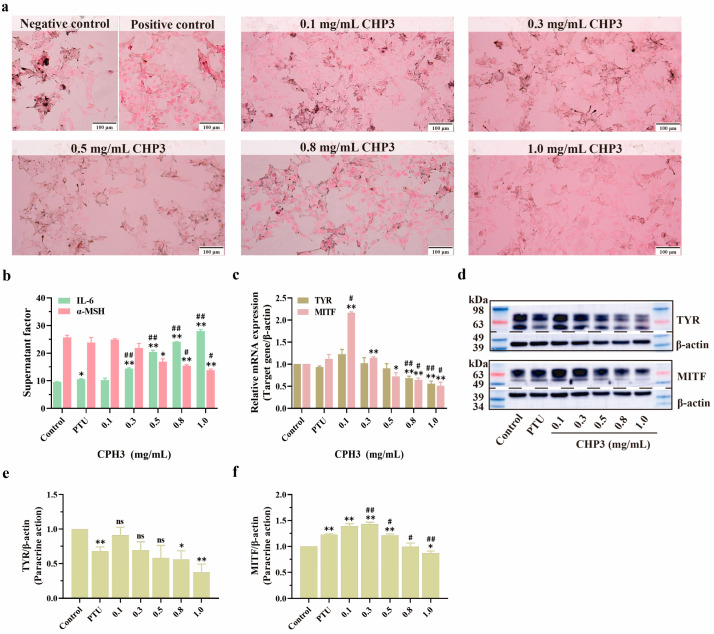
The effect of CPH3 on related protein expression in B16F10 cells under paracrine action. (**a**) In cultured B16F10, melanin pigmentation was visualized by Fontana–Masson staining. (**b**) The contents of factors IL-6 and α-MSH in HaCaT cell supernatant were detected by ELISA. (**c**) Densitometric analysis shows the relative intensity of the nuclear TYR and MITF. (**d**–**f**) Western blotting of TYR and MITF protein level in B16F10. * *p* < 0.05, ** *p* < 0.01, versus negative control group. # *p* < 0.05, ## *p* < 0.01, versus positive control group (PTU: 1-Phenyl-2-Thiourea, 50 μM), ns: no significant, *n* = 3.

Based on the preceding research findings, it is affirmed that type I collagen peptide derived from pig skin exerts a notable inhibitory effect on B16F10 melanin through HaCaT paracrine action, with CPH3 demonstrating a more pronounced inhibitory effect on melanin. Consequently, this section employed the Fontana–Masson staining method to directly observe the distribution of B16F10 melanin, as shown in Figure 5a. In the negative control group, melanin particles within B16F10 cells appeared darker and more agglomerated, indicative of higher melanin content. Conversely, in the positive control group treated with PTU, the color of intracellular melanin particles significantly lightened, and the melanin content reached its lowest level. Within the experimental group, as the concentration of CPH3 increased, the color of melanin particles in B16F10 cells progressively lightened.

In the human epidermis, keratinocytes and melanocytes are distributed in a ratio of approximately 36:1 [28]. While keratinocytes do not directly contribute to melanin generation, they regulate skin pigmentation through paracrine mechanisms. When stimulated by external factors, keratinocytes produce various factors that modulate melanin production, including the melanin suppressor interleukin-6 (IL-6) and the melanotropin α-melanocyte stimulating hormone (α-MSH).

IL-6 is a cytokine produced by keratinocytes. Studies have shown that the increase of IL-6 can down-regulate MITF factor in melanocytes and inhibit TYR activity [29,30]. Specific data showed that there was a significant dose-dependent relationship between CPH3 concentration and IL-6 and α-MSH expression. CPH3 stimulates keratinocytes to secrete more IL-6, which binds to a specific receptor (IL-6R), triggering a signaling cascade within the cell. So far, IL-6 is generally able to regulate MITF factor levels through the JAX-STAT (signal transduction and transcriptional activator) pathway, the MAPK (mitogen-activated protein kinase) cascade, and the PI3K (phosphatidylinositol 3-kinase) cascade [31,32]. Among them, MAPK and PI3K signaling pathways are important pathways in melanin production, controlling the synthesis of melanin, the formation of melanosomes, and the differentiation of black cells. α-MSH is produced by keratinocytes and binds to melanocortin 1 receptor (MC1R), which is specifically present in melanocytes and can activate adenosine cyclic phosphorus (cAMP) response element binding protein (CREB). The activated CREB may further regulate the transcription of downstream genes and upregulate the expression of the MITF factor. Furthermore, tyrosinase activity is enhanced, and melanin production is stimulated [33,34,35]. Figure 5b illustrates the IL-6 and α-MSH content in the supernatant of HaCaT cells. With increasing CPH3 concentration, IL-6 exhibits an upward trend, whereas α-MSH demonstrates the opposite trend. This compellingly suggests that collagen peptides inhibit melanin production via paracrine effects in keratinocytes. To further elucidate the mechanism of action of collagen peptides, the mRNA and protein expression trends of TYR and MITF in B16F10 cells were investigated under the paracrine influence of different concentrations of CPH3. As depicted in Figure 5c, as the CPH3 concentration increases, the mRNA levels of TYR and MITF significantly decrease, reaching 55.77 ± 6.09% and 50.70 ± 8.18% of the negative control group, respectively. This observation aligns with the trends observed in intracellular TYR and MITF protein expression via Western blotting (Figure 5d–f), original blots/gels are presented in Supplementary Figure S3.

### 3.6. Effects of CPH3 on Pigmentation Model in Zebrafish

Zebrafish commence melanin formation within 24 h of embryonic development, and between 24 and 72 h, their tissues and organs gradually develop, resulting in a transparent body transitioning to a black hue throughout. This presents an efficient, convenient, and cost-effective means for researchers to delve deeper into the mechanisms underlying melanin generation and deposition.

**Figure 6:**
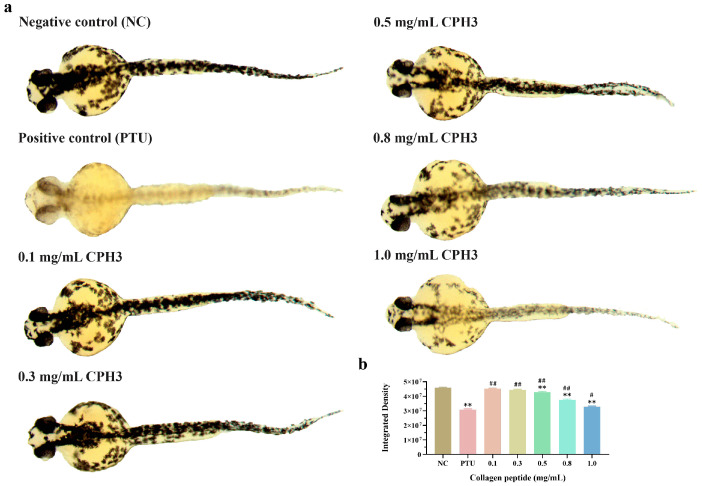
Effect of CPH3 on melanin in zebrafish juveniles. (**a**) The distribution of melanin in the body of zebrafish juveniles. (**b**) Analysis of intensity grayscale values of zebrafish melanin., ** *p* < 0.01, versus negative control group. # *p* < 0.05, ## *p* < 0.01, versus positive control group (PTU: 1-Phenyl-2-Thiourea, 50 μM), *n* = 10.

As depicted in Figure 6a, the ability to generate melanin in zebrafish gradually enhances with increasing CPH3 concentration. At a CPH3 concentration of 1.0 mg/mL, the inhibitory capacity against melanin reaches its peak strength. While the inhibitory effect on melanin is somewhat smaller than that of the PTU-positive control group, CPH3 exhibits high biocompatibility and safety. It is gentle and nonirritating to the skin barrier, thereby reducing the likelihood of allergies, inflammation, and other adverse reactions. Figure 6b displays the grayscale intensity of the negative control, positive control, and CPH3 (1.0 mg/mL), which were measured as 4.58 × 10^7^ ± 5.00 × 10^5^%, 3.07 × 10^7^ ± 6.91 × 10^5^% and 3.27 × 10^7^ ± 5.36 × 10^5^%, respectively. These results indicate that CPH3 maintains a potent inhibitory effect on zebrafish melanin generation.

### 3.7. Screening of CPH3 Tyrosinase Inhibitory Peptides

**Figure 7:**
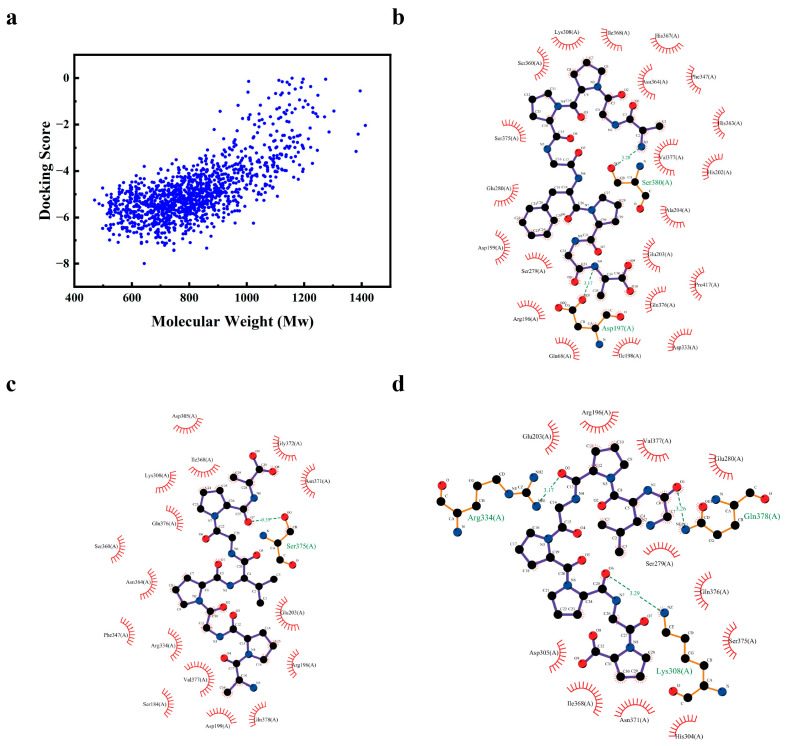
Screening of CPH3 tyrosinase inhibitory peptide. (**a**) Relationship between collagen polypeptides with binding capacity and molecular weights. (**b**) 2D diagram of molecular docking between TYR and Nona-AGA, (**c**) Octa-APA, and (**d**) Octa-GGP.

This study used the blind docking strategy to predict the inhibitory activity of 1351 collagen peptides and mTYR. Their binding free energy to mTYR was evaluated by AutoDock Vina to measure the binding affinity between ligands and receptors. Generally speaking, the lower the binding free energy, the more stable the bond between molecules is. The docking results are shown in Figure 7a, there are 1275 collagen peptides with binding energy less than 0. Among them, the three peptides with the stronger binding ability to mTYR are the Nona-AGPPGFPGA (Nona-AGA), Octa-APGPVGPA (Octa-APA), and the Octa-GLPGPPGP (Octa-GGP). It was further found through LigPlus software, as shown in Figure 7b, that Nona-AGA has a binding affinity with mTYR of −7.999 kJ/mol, forming two hydrogen bond interactions with Asp197 and Ser380. It also forms 21 hydrophobic interactions with Gln68, Arg196, Ile198, Asp199, His202, Glu203, Ala204, Ser279, Glu280, Lys308, Asp333, Phe347, Ser360, His363, Asn364, His367, Ile368, Ser375, Gln376, Val377 and Pro417. As shown in Figure 7c, the binding energy of Octa-APA with mTYR is −6.986 kJ/mol, forming a hydrogen bond interaction with Ser375. With Ser184, Arg196, Asp199, Glu203, Asp305, Lys308, Arg334, Ser360, Asn364, Ile368, Asn371, Gly372, Gln376, Val377 and Gln378 form 16 hydrophobic interactions. The binding energy of Octa-GGP with mTYR is −5.642 kJ/mol, forming three hydrogen bond interactions with Lys308, Arg334, and Gln378. With Arg196, Glu203, Ser279, Glu280, His304, Asp305, Ile368, Asn371, Ser375,Gln376 and Val377 form 11 hydrophobic interactions, as shown in Figure 7d. Through molecular docking results, we could determine that the inhibition of TYR by collagen polypeptide was a non-competitive inhibitor. 

### 3.8. Inhibitory Effects of Nona-AGA, Octa-APA and Octa-GGP on Melanin

**Figure 8:**
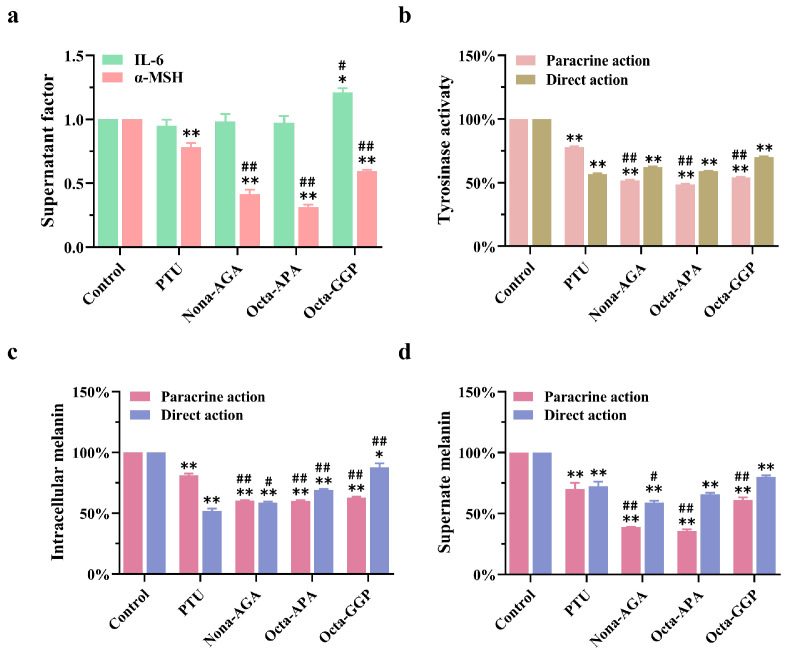
Inhibition of melanogenic activity of peptides in B16F10 cells. (**a**) Determination of factor content in IL-6 and α-MSH supernatant by Nona-AGA, Octa-APA, and Octa-GGP, effects of (**b**) TYR activity in B16F10 cells, and melanin content in (**c**) cells and (**d**) supernatant, the concentration of collagen polypeptide was 1.0 mg/mL. * *p* < 0.05, ** *p* < 0.01, versus negative control group. # *p* < 0.05, ## *p* < 0.01, versus the positive control group (PTU: 1-Phenyl-2-Thiourea, 50 μM), *n* = 3.

Figure 8a shows the effects of Nona-AGA, Octa-APA, and Octa-GGP (HPLC and LC-MS characterizations as shown in Supplementary Figure S4) on the levels of paracrine factors IL-6 and α-MSH. Among them, three collagen polypeptides could inhibit α-MSH secretion from HaCaT, while the overall significant difference in IL-6 secretion was not significant. Figure 8b shows the effects of three peptides on TYR activity in B16F10 cells under paracrine and direct action. The results showed that the three peptides could significantly reduce TYR activity under the two action pathways, and the paracrine effect was better. Finally, Figure 8c,d show the determination of intracellular and extracellular melanin content of three collagen peptides under two pathways of action. The results showed that Nona-AGA, Octa-APA, and Octa-GGP had a double effect on the inhibition of melanin content in B16F10 cells. Under paracrine action, the contents of intracellular melanin were 60.38 ± 0.48%, 60.14 ± 0.72%, and 62.77 ± 0.86%. Under direct action, the intracellular melanin contents were 58.63 ± 1.08%, 69.05 ± 0.88%, and 87.71 ± 3.35%, respectively. At the same time, the supernatant melanin content of Nona-AGA, Octa-APA, and Octa-GGP was 38.57 ± 0.10%, 35.58 ± 1.53% and 60.94 ± 2.22% in the paracrine effect, 59.09 ± 1.28%, 65.67 ± 1.37% and 80.10 ± 1.10% in the direct effect. These results reveal that they have great potential to become the next generation of TYR inhibitors.

## 4. Discussion

The hydrolysis of collagen involves the cleavage of protein peptide bonds, a process achievable through enzymatic, photothermal, and chemical reagent methods. Enzymatic hydrolysis, conducted under mild conditions, maximizes the preservation of collagen’s original biological activity. However, collagen peptides produced via enzymatic hydrolysis often necessitate inactivation or purification at high temperatures to prevent enzyme-related contamination, significantly elevating production costs. While research demonstrates that collagen peptides derived from enzymatic hydrolysis of animal skin exhibit antioxidant activity [36], their molecular weight typically ranges from several thousand to several hundred thousand, limiting their suitability for dermatological applications.

Park et al. [37] pioneered a method to directly extract collagen peptides with varying molecular weights from fish skin using high temperatures (150–250 °C) and high pressures (350–3900 kPa). By grading the hydrolysis products through ultrafiltration membranes, they found that components with molecular weights < 1 kDa exhibited the highest antioxidant activity, whereas those with 5–10 kDa showed the highest anti-aging activity. Although this approach enhances collagen-peptide yield, it demands substantial energy consumption during preparation. Alternatively, gene coding allows for the construction of specific collagen-peptide sequences using *Escherichia coli* [38,39]. However, this method entails stringent experimental conditions, high extraction costs, and limited yields. The collagen-peptide extraction method proposed in this study offers simplicity, speed, and cost-effectiveness. By adjusting the H+ concentration, researchers can obtain collagen peptides with lower molecular weights, facilitating further research and development.

Studies have shown that the DNA similarity between pigs and humans is as high as 98%. The similarity in genes implies that the amino acid sequence and intermolecular interactions of porcine skin-derived collagen peptides are more similar to those of human collagen, thereby more effectively exerting their biological functions [40,41]. Choi et al. [42] isolated small-molecular-weight hydrolyzed collagen peptides from porcine skin using hydrothermal (MW: 4270–20,100 Da) and ultrafiltration (MW: 985–20,100 Da) methods. These peptides demonstrated excellent absorption characteristics in the Franz cell model, with both achieving a clearance rate of 80% for 2, 2′-azino-bis(3-ethylbenzothiazoline-6-sulfonic acid (ABTS) free radicals. The inhibitory effects on tyrosinase were 33.56 ± 1.162% and 26.85 ± 3.487%, respectively. Similarly, Hong et al. [43] utilized alkaline protease to extract whole collagen peptides from pig by-products, resulting in a TYR inhibition rate of 15.44%. Plus, when the molecular weight was below 3 kDa, the TYR inhibition rate reached 30.2%. Other studies have also shown that compared with high-molecular-weight hydrolysis products, low-molecular-weight hydrolysis products possess stronger biological activities, such as antioxidant and tyrosinase inhibition [44,45]. Therefore, CPH1 and CPH3, which have a large difference in molecular weight, were selected to compare the inhibitory effect on melanin. As the results showed, the smaller CPH3 collagen peptide (MW: 500–1500 Da) inhibited melanin and tyrosinase in B16F10 cells better than CPH1 (MW: 778–3027 Da). After CPH3 direct treatment, the melanin content and TYR activity in B16F10 cells were 85.52 ± 0.83% (versus normal control) and 92.09 ± 1.17% (versus normal control), respectively, which were better than CPH1 (melanin content: 91.94 ± 1.81%, TYR activity:108.60 ± 0.93%).

In addition to pigskin-derived peptides, collagen derived from other species has also been shown to have melanin-inhibiting effects. Previous studies have shown that tyrosinase-inhibiting peptides derived from sea cucumber collagen effectively inhibited melanin production and reduced the occurrence of related skin diseases [46]. The application of grass carp fish-scale collagen peptide FTGML significantly inhibited tyrosinase activity and melanin synthesis in B16F10 melanoma cells [47]. Hou et al. [48] isolated collagen peptides with a molecular weight of 2–6 kDa from Pacific cod skin, which decreasing melanin content in B16 cells to 60.5% of the control group. Although these collagen peptides show inhibitory effects on melanin, the effects on melanocytes that have been studied are all direct actions. It is important to note that when collagen peptides interact with the skin, those with smaller molecular weights are more likely to penetrate the stratum corneum. Consequently, collagen peptides primarily interact with keratinocytes upon entering the epidermis, with only a minimal amount reaching the basal layer to influence melanocytes directly. Therefore, investigating the paracrine role of keratinocytes is crucial for understanding melanin secretion. This study revealed that when CPH3, characterized by a smaller molecular weight, directly interacts with B16F10 cells, the intracellular melanin content measures 85.82 ± 0.83% versus control. However, under paracrine action, this content significantly decreases to 65.23 ± 1.30% versus control. Both direct and indirect action substantially enhances CPH3’s ability to inhibit melanin production. In this study, it was found that CPH3 inhibited the level of MITF factor in B16F10 cells by enhancing IL-6 and decreasing α-MSH in HaCaT cells, thereby reducing TYR activity and ultimately inhibiting the production of melanin.

Zhuang et al. [49] extracted the 161 tyrosinase inhibitory peptide from the Sipunculus nudus gelatin hydrolysate and screened the biological activity through molecular docking. Hu et al. [50] combined bioaffinity ultrafiltration and LC-Orbitrap-MS/MS technology to successfully screen TYR inhibitory peptides in the hydrolysate of grass carp fish-scale gelatin and screened four new TYR inhibitors from the hydrolysate through molecular docking. Xue et al. [17] identified three TYR activity-inhibiting tripeptides from donkey collagen, including Asp-Gly-Leu, Gly-Ala-Arg, and Ser-Asp-Trp. The IC_50_ values were 0.47 ± 0.01 mM, 1.13 ± 0.04 mM, and 2.08 ± 0.01 mM, respectively. Based on the excellent melanin inhibition ability of CPH3, the homologous model of mouse tyrosinase (mTYR) was constructed by YASARA software in this study, and a database of interaction between collagen polypeptide and mTYR was constructed, and 1275 collagen polypeptide fragments with inhibitory effect on mTYR activity were efficiently screened out. Represented by Nona-AGA, Octa-APA, and Octa-GGP, the inhibitory effects of these three collagen peptides on melanin were evaluated through in vitro cell experiments. The results showed that Nona-AGA, Octa-APA, and Octa-GGP inhibited TYR activity and melanin production in B16F10 cells through direct and indirect actions, Nona-AGA and Oc-ta-APA had better inhibitory effects on melanin production than Octa-GGP.

## 5. Conclusions

This study focuses on the extraction and bioactivity of collagen polypeptides and their application in inhibiting melanin production. We successfully achieve the extraction of collagen peptides with a molecular weight of 0.3 to 1.5 kDa using an economically efficient acid hydrolysis method. Our findings demonstrate that CPH3 exhibits the ability to down-regulate MITF factors in melanocytes through HaCaT paracrine action, thereby reducing TYR activity and inhibiting melanin production. Through amino acid sequence analysis, the potential peptides were further screened, and their inhibitory effects on melanin were verified by in vitro cell experiments.

In this study, the inhibitory effect of collagen peptide on melanin was studied from three aspects: in vitro evaluation model, cell level evaluation model, and animal level evaluation model. These data provide us with valuable research information, but it is necessary to carefully consider physiological differences, drug metabolism, immune system response, and other factors when applying these results to humans. Through reasonable experimental design and multidisciplinary research methods, the translatability of in vitro experimental results can be improved to provide more effective support for human health and disease treatment. Moving forward, we will conduct further in vitro and in vivo studies, clinical trials, and the development of new products based on CPH3 collagen polypeptides.

## Figures and Tables

**Table 1:** The effects of CPH1 and CPH3 on melanin and TYR.

1 mg/mL		Supernatant Melanin	*p* Value	Intracellular Melanin	*p* Value	Tyrosinase Activity	*p* Value
Paracrine action	CPH1	94.34 ± 0.54%	*	81.84 ± 1.41%	***	85.53 ± 1.66%	***
	CPH3	82.96 ± 3.07%		65.23 ± 1.30%		61.50 ± 1.15%	
Direct action	CPH1	106.17 ± 3.56%	ns	91.94 ± 1.81%	*	108.60 ± 0.93%	***
	CPH3	103.65 ± 4.49%		85.52 ± 0.83%		92.09 ± 1.17%	

* *p* < 0.05, *** *p* < 0.001, ns: no significant, *n* = 3.

## Data Availability

The original contributions presented in this study are included in the article/Supplementary Materials. Further inquiries can be directed to the corresponding author(s).

## Supplementary Materials

The following supporting information can be downloaded at: https://www.mdpi.com/article/10.3390/biom15020220/s1. Figure S1: SDS-PAGE analysis of type I collagen from pig skin; Figure S2. The effects of CPH2 on melanin and TYR; Figure S3. Original Western blot data in the main article; Figure S4. The characterization of Nona-AGA, Octa-APA and Octa-GGP.
