# Supplementary material for: The Inhibitory Effect of Peptide Hydrolysate of Type I Collagen Derived from Pig Skin on Melanogenesis in B16F10 Melanoma Cells
Source: Biomolecules. 2025 Feb 3;15(2):220. doi: 10.3390/biom15020220 (PMC11852596; doi:10.3390/biom15020220)
Supplement: Supplementary file 1 [file biomolecules-15-00220-s001.zip › biomolecules-3369036-supplementary.pdf]

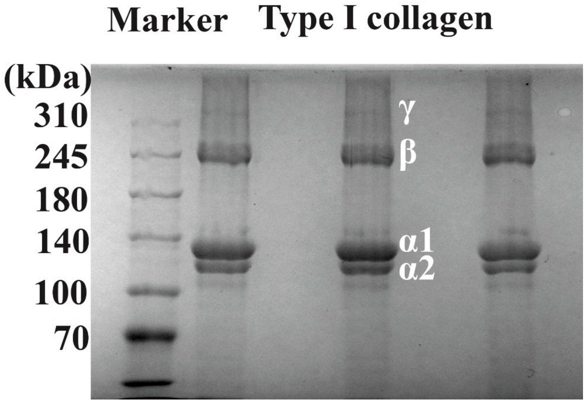

Supplementary Figure S1: SDS - PAGE analysis of type I collagen from pig skin.

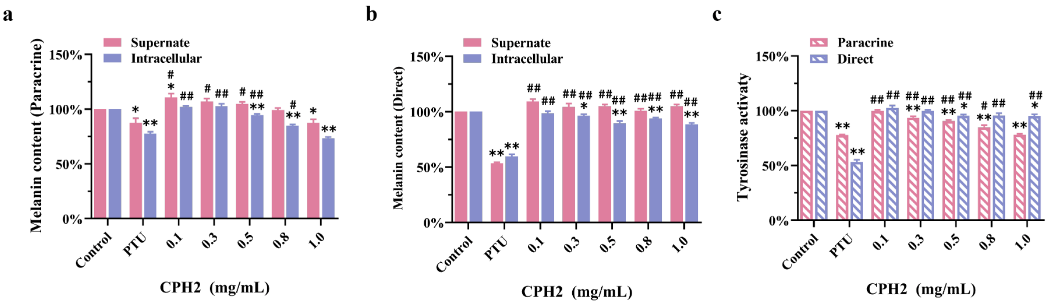

Supplementary Figure S2. The effects of CPH2 on melanin and TYR. (a) The content of melanin in B16F10 under paracrine pathway. (b) The content of melanin in B16F10 under direct pathway. (c) TYR activity in B16F10 under paracrine and direct pathway. *P<0.05, **P<0.01, versus negative control group. #P<0.05, ##P<0.01, versus posi-tive control group (PTU: 1-Phenyl-2-Thiourea, 50 μM), *n*=3.

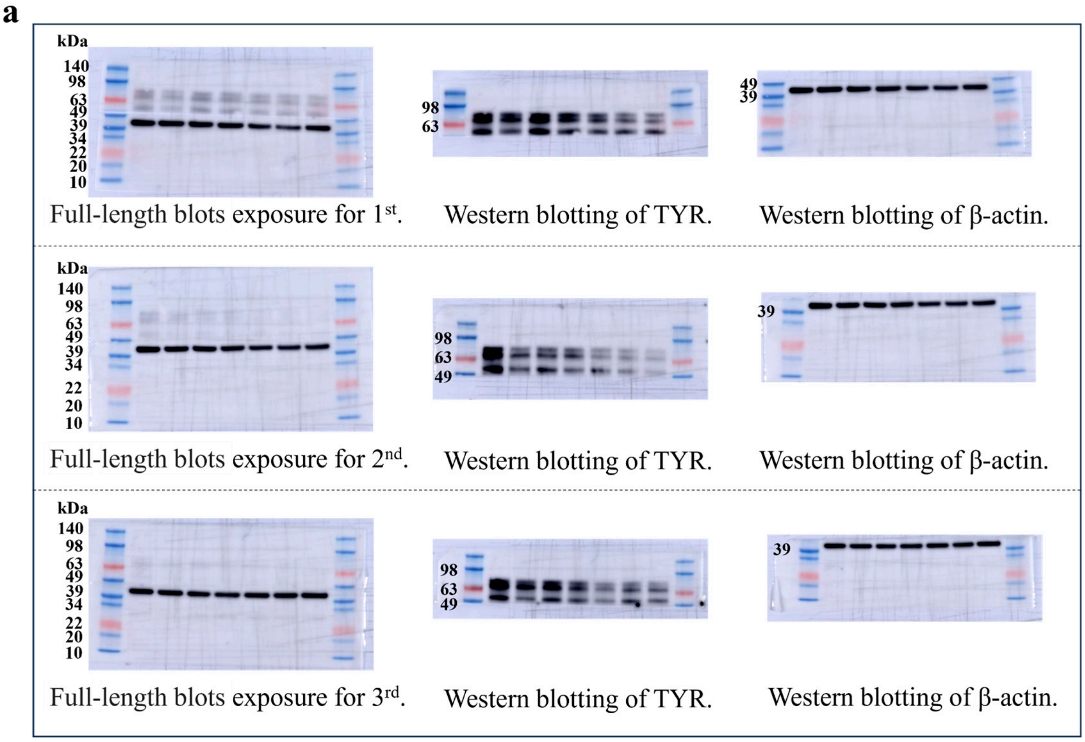

b

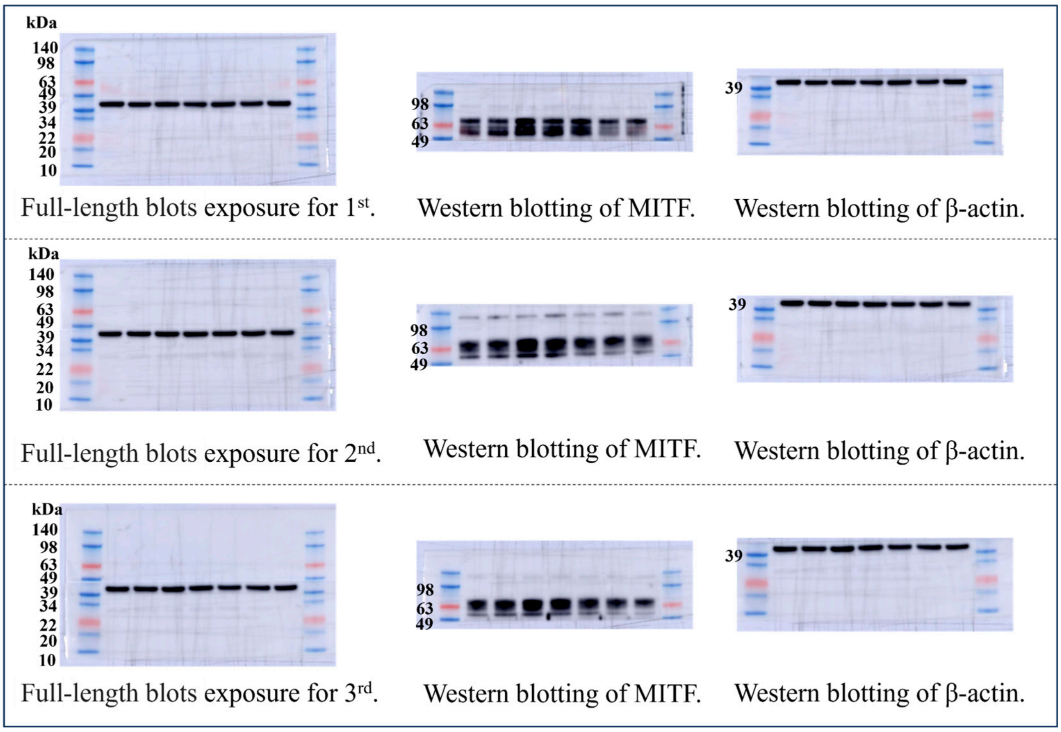

Supplementary Figure S3. Original Western blot data in the main article. (a) Western blotting of TYR/β-actin. (b) MITF/β-actin protein level in B16F10. TYR predicted band size: 50-80 kDa. MITF predicted band size: 50-70 kDa. β-actin predicted band size: 42 kDa.

Supplementary Figure 3(a) shows the HPLC chromatograms of Nona-AGA, Octa-APA, and Octa-GGP. The relative contents of each component were calculated by the normalization method. The retention time of the target compound Nona-AGA is 10.456 min, and the peak area is 95.739 relative units; the retention time of Octa-APA is 11.256 min, and the peak area is 98.774 relative units; the retention time of Octa-GGP is 8.822 min, and the peak area is 98.382 relative units. The purity of the compounds is relatively high, meeting the experimental requirements. Supplementary Figure 4(b) shows the MS spectra of Nona-AGA, Octa-APA, and Octa-GGP. The mass-to-charge ratio (m/z) ranges from 200 to 2000, covering those of the expected compounds and their possible fragment ions in the sample. In the positive ion mode, the quasi-molecular ion peak $[M+H]^+$ of Nona-AGA was observed with a mass-to-charge ratio of 770.30. It can be inferred that the molecular weight of this compound is approximately 769.30, which is consistent with the theoretical molecular weight of 769.90. The quasi-molecular ion peak $[M+H]^+$ of Octa-APA has a mass-to-charge ratio of 665.35. It can be inferred that

the molecular weight of this compound is approximately 664.35, which is consistent with the theoretical molecular weight of 664.80. The quasi-molecular ion peak $[M+H]^+$ of Octa-GGP has a mass-to-charge ratio of 691.40. It can be inferred that the molecular weight of this compound is approximately 690.40, which is consistent with the theoretical molecular weight of 690.80.

a

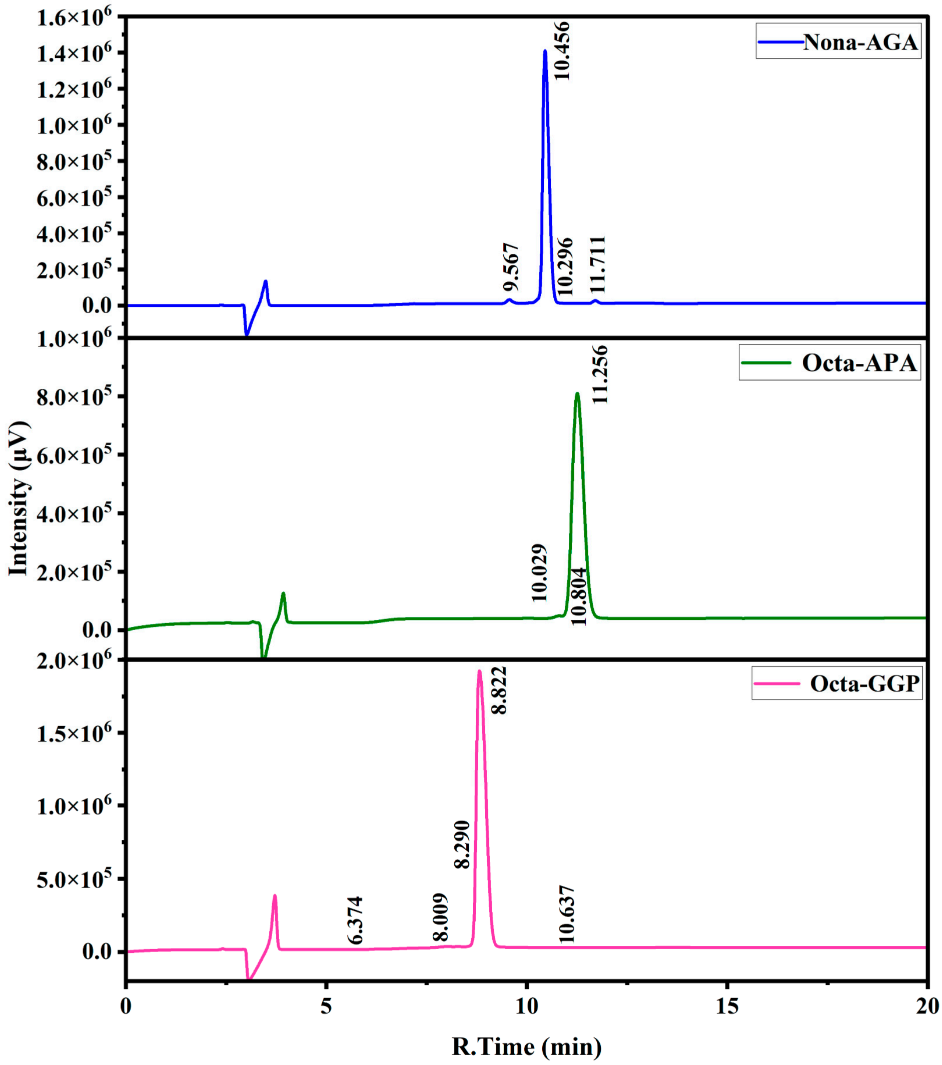

b

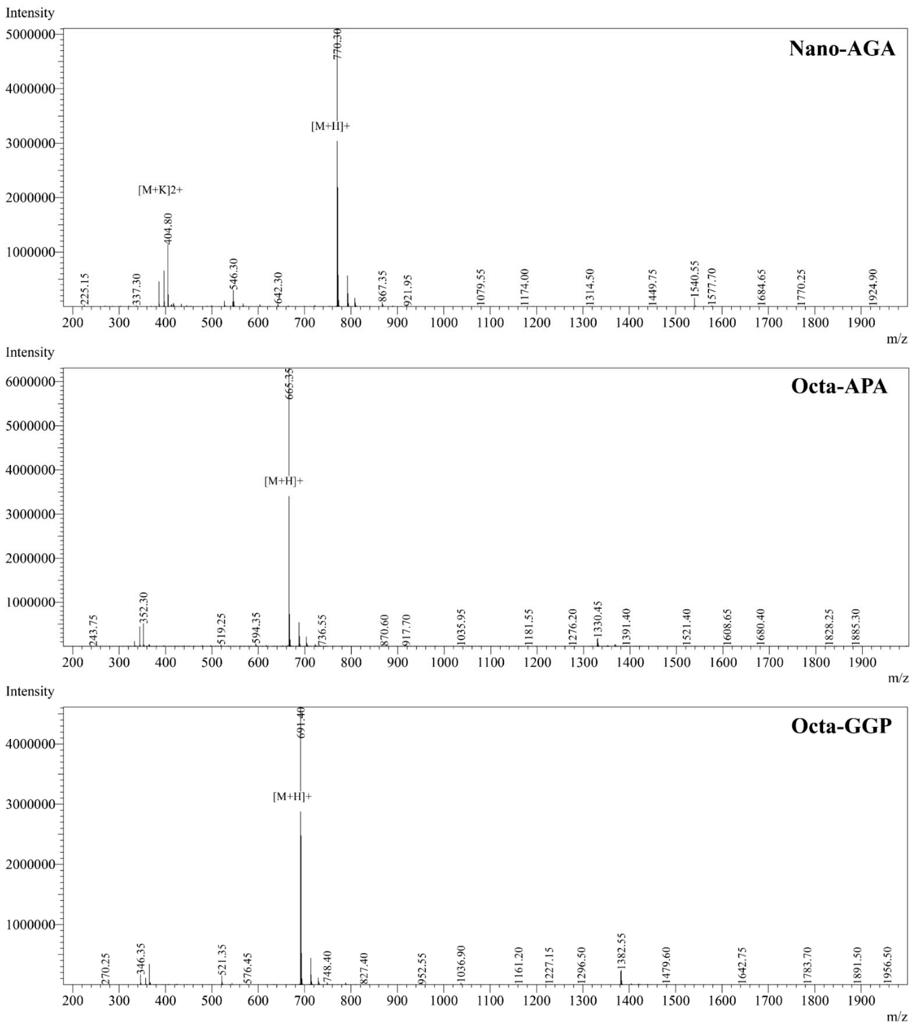

Supplementary Figure S4. The characterization of Nona-AGA, Octa-APA and Octa-GGP. (a) HPLC of Nona-AGA, Octa-APA and Octa-GGP, the purity > 98%. (b) LC-MS of Nona-AGA, Octa-APA and Octa-GGP.
